# Effectiveness of the hypoxic exercise test to predict altitude illness and performance at moderate altitude in high‐level swimmers

**DOI:** 10.14814/phy2.14390

**Published:** 2020-04-23

**Authors:** Robin Pla, Franck Brocherie, Sébastien Le Garrec, Jean‐Paul Richalet

**Affiliations:** ^1^ Laboratoire Sport Expertise et Performance Institut National du Sport (INSEP) Paris France; ^2^ Institut de Recherche Médicale et de bio‐Epidémiologie du Sport (IRMES) Paris France; ^3^ Fédération Française de Natation (FFN) Clichy France; ^4^ Département Médical Institut National du Sport (INSEP) Paris France; ^5^ UMR INSERM 1272 Hypoxie et poumon Université Paris 13 Bobigny France

**Keywords:** altitude, elite athletes, testing

## Abstract

**Purpose:**

The hypoxic exercise test is used to predict the susceptibility to severe High Altitude Illness (SHAI). In the present study, we aimed to use this test to predict the changes in performance and the physiological responses to moderate altitude in elite swimmers.

**Methods:**

Eighteen elite swimmers performed a hypoxic exercise test at sea level before a moderate 12‐day altitude training camp (1,850 m) to determine if they were susceptible or not to SHAI. A maximal swimming performance test was conducted before (at sea level), during (at 1,850 m), and after (at sea level) the intervention. Arterial oxygen saturation (pulse oximetry), Lake Louise score, and quality of sleep questionnaire were collected every morning. The participants were classified in two groups, those who had a moderate to high risk of SHAI (SHAI_score_ ≥ 3) and those who had a low risk of SHAI (SHAI_score_ < 3).

**Results:**

Seven swimmers presented a high risk of SHAI including three of them with a SHAI_score_ > 5. Pearson correlations indicated that SHAI_score_ was strongly correlated with the decrease in swimming performance at altitude (*r* = .60, *p* < .01). Arterial oxygen saturation during the hypoxic exercise test was the physiological variable that was best related to performance decrease at altitude (*r* = .54, *p* < .05). No differences were observed for Lake Louise score and quality of sleep between swimmers who suffered from SHAI or not (*p* > .1).

**Conclusion:**

In a population of elite swimmers, the combination of clinical and physiological variables (SHAI_score_, oxygen desaturation) estimated the performance decrease at moderate altitude. The hypoxic exercise test could allow coaches and scientists to better determine the individual response of their athletes and manage the altitude acclimatization.

## Graphical abstract

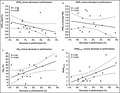

## 1 INTRODUCTION

Since the 1968 Olympic Games in Mexico City, the interest in altitude/hypoxic training (hypobaric or normobaric) for elite athletes has strongly increased. Most of the best endurance athletes regularly dedicate part of their training at altitude (Wilber, 2007). Today, it is well‐accepted that chronic hypoxic exposure may induce physiological acclimatization, such as a higher concentration of hemoglobin, an increase in erythrocyte volume, a greater maximal aerobic exercise capacity and/or working economy, that would enhance aerobic performance using legal ergogenic aid (Sinex & Chapman, 2015).

During the first days of hypoxic exposure, some detrimental effects can occur such as a decrease in maximal oxygen consumption, requiring to appropriately reduce and manage training loads (Brocherie et al., 2015; Brocherie, Schmitt, & Millet, 2017; Issurin, 2010). Several studies have shown a large interindividual variability in the physiological responses to prolonged hypoxic exposure associated with training (Chapman, Stray‐Gundersen, & Levine 1998; Millet, Roels, Schmitt, Woorons, & Richalet, 2010) Changes in some physiological variables (i.e., an increase in ventilatory response; Boron & Boulpaep, 2012, a decrease in plasma volume; Sawka, Convertino, Eichner, Schnieder, & Young, 2000, or an increase in erythropoietin production; Lundby et al., 2007) may differ between athletes after few days of training in hypoxia.

Therefore, it would be interesting to evaluate these individual responses to hypoxic exposure, before initiating a process of altitude acclimatization or a training camp. Until now, some authors described hypoxic tests to identify the individual risk of severe high‐altitude illnesses (SHAI; Canoui‐Poitrine et al., 2014; Gibson, Richardson, Hayes, Duncan, & Maxwell, 2015; Richalet, Larmignat, Poitrine, & Letournel, 2012). Richalet's research group demonstrated that some physiological responses observed during a hypoxic exercise test (i.e., low ventilatory and cardiac responses to hypoxia at exercise, high desaturation during exercise) were strongly correlated with a higher risk of SHAI in a large population of altitude visitors (Canoui‐Poitrine et al., 2014; Richalet et al., 2012). Gibson et al. (2015) used a 6‐min walk test in hypoxia to predict physiological responses to exercise in hypoxia in healthy participants. This study suggested that changes in heart rate and post‐test oxygen saturation might be useful to determine the quality of acclimatization to altitude, but these variables were not able to predict performance. However, none of these studies were conducted with elite athletes.

Therefore, the purpose of this study was to assess if the hypoxic exercise test previously developed by Richalet et al. (2012) for identifying SHAI risk would be appropriate to predict performance and tolerance at moderate altitude (1,850 m) in elite swimmers. It was hypothesized that the combination of clinical and physiological parameters determined during the Richalet's test would predict variations in performance at moderate altitude.

## 2 MATERIALS AND METHODS

### 2.1 Participants

This study included 18 elite swimmers from the French Swimming Federation. The physical and performance characteristics of those swimmers are presented in Table 1. The study was approved by the local ethics committee and conducted in accordance with the Declaration of Helsinki. After comprehensive verbal explanations, all participants signed an informed consent form to participate.

**Table 1:** Characteristics of the population studied

Parameter	Mean ± *SD*
Age	18.6 ± 1.1
Height (m)	1.77 ± 0.09
Body mass (kg)	67.4 ± 7.4
BMI (kg/m^2^)	21.3 ± 1.4
Fat mass (%)	13.3 ± 5.8
Percentage of world record (%)	90.40 ± 1.66

Note

Percentage of world record was based on the event performed by each swimmer.

Abbreviation: BMI, body mass index.

### 2.2 Experimental overview

The study design consisted of: (a) a hypoxic exercise test before a moderate altitude training camp, (b) three successive swimming performance tests conducted (i) at sea‐level 5 days before (pre‐test) initiating a 12‐day training camp at moderate altitude (Font‐Romeu, France, 1,850 m); (ii) at 1,850 m after 8 days of altitude training, with the same training protocol for each swimmer (see training assessment subsection); and (iii) at sea level 3 days after (post‐test) the end of the altitude training camp.

### 2.3 Richalet's hypoxic exercise test

**Figure 1:**
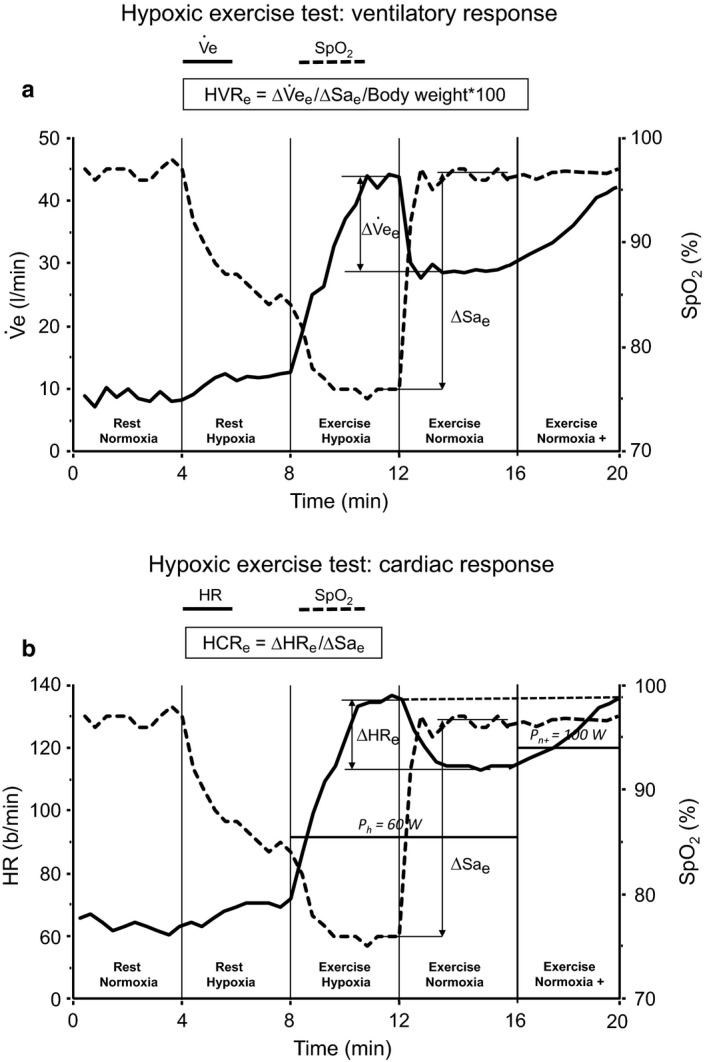
Richalet's hypoxic exercise test. (a) Ventilatory response to hypoxia; (b) cardiac response to hypoxia. HR, heart rate; Ph and Pn+, power output at exercise in hypoxia and in normoxia for the same value of heart rate than in hypoxia; SpO2, arterial oxygen saturation; Ve, minute ventilation (adapted from Richalet et al., 2012, 2015)

During the week preceding the altitude training camp, all the swimmers performed the hypoxic exercise test. This test was previously described by Richalet et al. (2012) and Richalet, Lhuissier, Larmignat, and Canouï‐Poitrine (2015). Briefly, swimmers performed a submaximal ergocycle exercise test (30% of maximal oxygen consumption measured in normoxia) in hypoxia (oxygen inspired fraction (FIO_2_) = 0.115 and normoxia (FIO_2_ = 0.209; Figure 1). From this test, the cardiac response to hypoxia at exercise (HCR_e_), the ventilatory response to hypoxia at exercise (HVR_e_) and the decrease in arterial O_2_ saturation at exercise in hypoxia (ΔSa_e_) were calculated. Then an overall score (SHAI_score_) – which allows detection of individuals prone to develop SHAI when exposed to high altitude (Canoui‐Poitrine et al., 2014; Richalet et al., 2012) – was computed. As previously described (Canoui‐Poitrine et al., 2014; Richalet et al., 2012, 2015), variables used to compute the score are age, gender, history of migraine and/or SHAI, regular endurance training, HCR_e_, HVR_e_, and ΔSa_e_). A SHAI_score_ ≥ 5 is associated with severe acute mountain sickness during high‐altitude exposure (Canoui‐Poitrine et al., 2014). In this study performed at moderate altitude, the participants were classified in two groups, those who had a SHAI_score_ < 3 and of those who had a SHAI_score_ ≥ 3.

### 2.4 Performance tests

The time‐trial performance test consisted for each swimmer to realize his specialty event (either 100 or 200 m) after a standardized 1,200 m warm‐up. A delay of 30 min between warm‐up and time‐trial performance test was allowed to the swimmers. Each test was performed at 4.00 p.m. to avoid any circadian variation. Change in performance was calculated in percentage of the pretest value (expressed in swimming speed).

### 2.5 Training assessment

All swimmers followed the same training program during the entire study period. As such, training content was the same for each swimmer in order to compare the individual differences from the hypoxic exercise test. Training consisted of 11 ± 1 sessions of swimming per week, corresponding to 74 ± 9 km weekly and 3 ± 1 sessions of strength conditioning per week from the pretest to the post‐test.

After each training session, rating of perceived exertion (RPE) was collected using the 6 to 20 Borg scale (1982).

### 2.6 Daily physiological measures and questionnaires

During the study period, arterial oxygen saturation was recorded in standing position using fingertip pulse oximeters (CMS, Contec), every morning upon waking up, just before breakfast. During breakfast, all participants were required to complete two questionnaires: (a) the Lake Louise questionnaire, which included five questions that were described as sensitive to quantify acute mountain sickness ( Roach, Bartsch, Hackett, & Oelz, 1993). Overall Lake Louise score was calculated by summing scores to the five questions; and (b) the 15‐item Groningen Sleep Quality Scale (GSQS), which was used to evaluate high‐altitude sleep disturbance (Weil, 2004).

### 2.7 Statistical analyses

The baseline characteristics of participants who had a SHAI_score_ < 3 and of those who had a SHAI_score_ ≥ 3 were compared using a Student's *t* test. Quantitative variables are presented as mean ± standard deviation (*SD*). Pearson's product‐moment correlations were used to examine the relations between the physiological variables measured during the hypoxic exercise test and the changes in performance (expressed as percentage of the pre‐test). The correlation coefficients (*r*) were interpreted in accordance with the following scale of magnitude (Hopkins, Marshall, Batterham, & Hanin, 2009): ≤0.1, trivial; >0.1–0.3, small; >0.3–0.5, moderate; >0.5–0.7, large; >0.7–0.9, very large; and >0.9–1.0, almost perfect. Null hypothesis was rejected at *p* < .05. Statistical analyses were undertaken using the software package STATISTICA (version 8.0, Statsoft).

## 3 RESULTS

### 3.1 Hypoxic exercise test

Table 2 describes the variables predicting SHAI, measured during the hypoxic exercise test for all participants. Differences in physiological parameters between the swimmers who showed a SHAI_score_ < 3 and those who presented a SHAI_score_ ≥ 3 were observed.

**Table 2:** Hypoxic exercise test results (SHAI < 3 versus SHAI ≥ 3)

Variable	All (18)	SHAI < 3 (*n* = 11) Mean ± *SD*	SHAI ≥ 3 (*n* = 7) Mean ± *SD*	*p*‐value
HCR_e_ (bpm/%)	1.11 ± 0.44	1.33 ± 0.42	0.75 ± 0.06	.002
HVR_e_ (L min^−1^ kg^−1 ^* 100)	1.34 ± 0.56	1.61 ± 0.55	0.91 ± 0.22	.006
ΔSa_e_ (%)	16.67 ± 5.73	13.45 ± 3.98	21.71 ± 4.23	.000
SHAI	2.61 ± 1.91	1.36 ± 0.45	4.57 ± 1.62	.000

Abbreviations: HCR_e_, cardiac response to hypoxia during exercise; HVR_e_, ventilatory response to hypoxia during exercise; ΔSa_e_, change in arterial oxygen saturation during exercise; SHAI, risk of severe high altitude illnesses.

**Figure 2:**
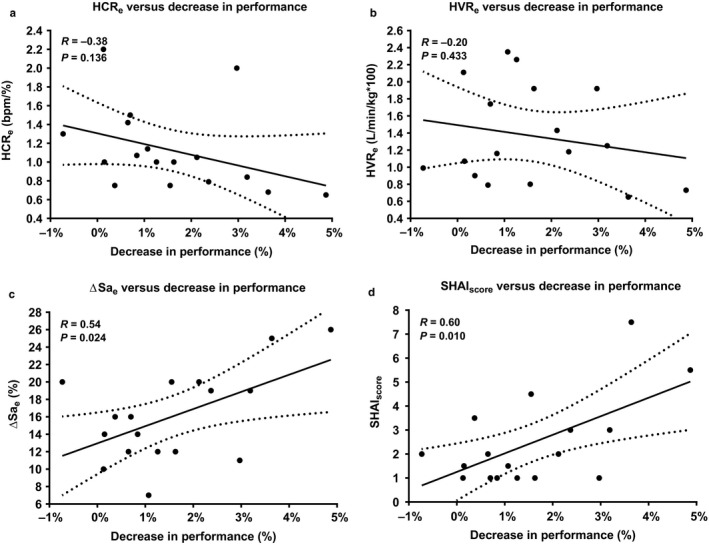
Correlations between cardiac response to hypoxia during exercise (a), ventilatory response to hypoxia during exercise (b), change in arterial oxygen saturation (c), risk of severe high‐altitude illnesses (d), and change in performance after 8 days of altitude training with 95% confidence intervals

Pearson's product‐moment correlations show that the SHAI_score_ was the variable the most correlated (*r* = .60, *p* = .01) to the change in performance at altitude (Figure 2). Three swimmers presented a SHAI_score_ < 5 with one of them injured during the altitude training camp while the two others obtained the poorest change in mean swimming performance (+3.64% and +4.87% respectively, see Figure 2e). Desaturation at exercise in hypoxia was also correlated with the change in performance at altitude (*r* = .54, *p* = .02). Other variables (cardiac and ventilatory responses) were poorly related to the change in performance (*r* = −.38, *p* = .136 and *r* = −.20, *p* = .433 respectively).

Table 3 provides the results of the hypoxic test, comparing the swimmers who had a performance decrease (at altitude) under 1.5% with those who had a performance decrease (at altitude) above 1.5%.

**Table 3:** Hypoxic exercise test results (PERF < 1.5% vs. Perf > 1.5%)

Variable	PERF_ALT_ < 1.5% (*n* = 9) Mean ± *SD*	PERF_ALT_ > 1.5% (*n* = 8) Mean ± *SD*	*p*‐value
HCR_e_ (bpm/%)	1.26 ± 0.42	0.97 ± 0.44	.179
HVR_e_ (L min^−1^ kg^−1^)	1.49 ± 0.63	1.24 ± 0.50	.382
ΔSa_e_ (%)	13.44 ± 3.78	19 ± 5.34	.025
SHAI	1.61 ± 0.82	3.44 ± 2.27	.039

Abbreviations: HCR_e_, cardiac response to hypoxia during exercise; HVR_e_, ventilatory response to hypoxia during exercise; PERF_ALT_, change in performance after 8 days of altitude training camp; ΔSa_e_, change in arterial oxygen saturation during exercise; SHAI, risk of severe high altitude illnesses.

### 3.2 Performance

After 8 days of altitude exposure, mean performance decreased by 1.58 ± 1.46%. Significant differences were observed between the SHAI_score_ < 3 and the SHAI_score_ ≥ 3 groups with a mean decrease in performance of 0.98 ± 1.00% and 2.67 ± 1.59%, respectively (Table 4). Three days after the end of the training camp, performances returned to near pre‐test values (mean performance change was decreased by 0.17 ± 0.78%). No significant change in performance was observed between the two groups.

**Table 4:** Performance at altitude results (SHAI < 3 versus SHAI ≥ 3)

Variable	SHAI < 3 Mean ± *SD*	SHAI ≥ 3 Mean ± *SD*	*p*‐value
PERF_ALT_ (%)	0.98 ± 1.00	2.67 ± 1.59	.017
SpO2 (%)	94.47 ± 0.93	93.55 ± 0.61	.035
RPE	13.71 ± 0.64	13.38 ± 0.43	.024
LAKE_1DAY_	1.64 ± 1.21	2.00 ± 1.41	.567
LAKE_3DAYS_	1.88 ± 0.64	1.91 ± 1.44	.953
SLEEP_1DAY_	3.09 ± 3.33	4.57 ± 3.21	.365
SLEEP_3DAYS_	3.88 ± 2.13	4.33 ± 2.52	.687
PERF_POST_ (%)	0.18 ± 0.88	0.14 ± 0.64	.923

Abbreviations: LAKE_1DAY_, Lake Louise score after the first night of altitude; LAKE_3DAYS_, Lake Louise score after three nights of altitude; PERF_ALT_, change in performance after 8 days of altitude training camp; RPE, mean of rating of perceived exertion for each training sessions during 8 days of altitude training camp; SLEEP_1DAY_, SLEEP score after the first night of altitude; SLEEP_3DAYS_, SLEEP score after three nights of altitude; SpO2, mean arterial oxygen saturation during 8 days of altitude training camp.

### 3.3 Arterial oxygen saturation

Mean arterial oxygen saturation during the altitude training camp was 94.1 ± 0.9%. On day 8, swimmers of the SHAI_score_ < 3 group had a higher arterial oxygen saturation than those from the SHAI_score_ ≥ 3 group (94.5 ± 0.9% versus 93.6 ± 0.6%, respectively, *p* < .05).

### 3.4 Perceived fatigue and questionnaires

No significant differences were observed for RPE, Lake Louise score and GSQS score between the two groups (Table 4). Mean Lake Louise score was 1.78 ± 1.26 after one night at 1,850 m, and 1.89 ± 0.98 after three nights at 1,850 m. Mean GSQS score was 3.67 ± 3.27 after one night at 1,850 m and 4.05 ± 2.23 after three nights at 1,850 m.

## 4 DISCUSSION

This study is the first to examine the effectiveness of the Richalet's hypoxic exercise test (Richalet et al., 2012, 2015) to determine performance responses and tolerance at moderate altitude in elite swimmers. The findings suggest that SHAI_score_ could be the best parameter – in comparison with the other physiological parameters investigated in this study – to predict the performance decrease at moderate altitude.

As already reported by Richalet et al. (2012) to predict SHAI, the hypoxic exercise test appears also useful to forecast performance response at moderate altitude. However, taken individually, cardiac or ventilatory responses do not seem to predict performance at altitude. Further studies investigating the responses and associations at various altitude levels are warranted to better understand the usefulness of each physiological variable. Only arterial oxygen desaturation during exercise was correlated with the changes in performance, as previously observed with trekkers (Richalet et al., 2012). The SHAI_score_ was designed for predicting high altitude tolerance, but it would also appear efficient to predict exercise tolerance at moderate altitude. These results also highlighted the large interindividual variability of physiological adaptations in elite swimmers in response to prolonged hypoxic exposure as already reported by Mounier et al. (2009) for blood markers.

It is interesting to note that no correlation was found between SHAI_score_ and Lake Louise score. This may be explained by the low hypoxic dose (training camp at 1,850 m) encountered by the swimmers. The altitude level was not high enough and arterial oxygen saturation was not low enough to significantly trigger severe forms of acute mountain sickness but were sufficient to have an impact on performance (Karinen, Peltonen, Kähönen, & Tikkanen, 2010; Roach, Greene, Schoene, & Hackett, 1998). As shown by Gore et al. (1997), maximal oxygen consumption is already reduced at low altitude (>500 m) and elite athletes are more sensitive than the general population for these changes. It is likely that swimmers who have only slightly reduced their performance – compared to others – have a greater ventilatory efficiency (Bernardi, Schneider, Pomidori, Paolucci, & Cogo, 2006). Further investigations would be required to determine the importance of ventilatory response to hypoxia on swimming performance at altitude.

After return at sea level, no difference in change of performance was observed between the two groups. This may indicate that the hypoxic exercise test is able to predict acute performance at altitude but is not appropriate to explain changes in performance at sea level after 12 days of moderate altitude training camp. In line with this, the results may reflect that performance measured at altitude is not directly related to the physiological adaptations and performance after return at sea level. Overall, the response to the hypoxic exercise test is indicative of performance responses and tolerance at moderate altitude. Although careful monitoring of training load is important during altitude acclimatization (Brocherie et al., 2015, 2017; Issurin, 2010), this highlights the particular attention that must be carried on athletes sensitive to hypoxic stress.

Some shortcomings should be pinpointed. First, the number of participants is rather limited and further investigations with a larger sample size are required to confirm the present results. Also, this study involved swimmers only. It would be necessary to observe whether these results will be similar with other sports. Moreover, only three participants were intolerant to severe hypoxia and presented a high risk of SHAI. Therefore, it would be interesting to observe the effects on performance at a higher altitude level. It is also important to note that the swimming performance test was measured after 8 days of altitude exposure, which allowed the swimmers to already improve their physiological capacities after this acclimatization period. Earlier testing (e.g., 2–3 days after arrival) would have probably reinforced the associations observed as expected performance would have been worse. Otherwise, even if the decrease in swimming performance at moderate altitude was correlated to the SHAI_score_, the results of the study do not evidence which physiological acclimatization could account for this higher decrease in “non‐responders”. Finally it could be difficult to reconcile a test validated for high altitude exposure with moderate altitude performance. However, the basic physiological mechanisms involved remain the same: the sensitivity of peripheral chemoreceptors that determine the ventilatory response to hypoxia and the level of desaturation at exercise, which are both determinants in the susceptibility to severe acute mountain sickness and to aerobic performance at altitude.

To conclude, the results of this study suggest that the Richalet's hypoxic exercise test may detect elite swimmers who would have difficulties to appropriately acclimatize to an altitude training camp. Furthermore, this may help coaches and background staffs to individualize the training load to each athlete, especially during the early phase of acclimatization when the hypoxic stress is the highest.

## CONFLICT OF INTEREST

The authors do not have any conflict of interest.
